# Pan-cancer analysis shows that IBSP is a potential prognostic and immunotherapeutic biomarker for multiple cancer types including osteosarcoma

**DOI:** 10.3389/fimmu.2023.1188256

**Published:** 2023-06-29

**Authors:** Boyu Pan, Xiaoyun Cheng, Wei Tan, Renfeng Liu, Xin Wu, Jinpeng He, Qizhi Fan, Yan Zhang, Jun Cheng, Youwen Deng

**Affiliations:** ^1^ Department of Spine Surgery, The Third Xiangya Hospital, Central South University, Changsha, Hunan, China; ^2^ Department of Pulmonary and Critical Care Medicine, The Third Xiangya Hospital of Central South University, Changsha, Hunan, China; ^3^ Department of Respiratory Medicine, Xiangya Hospital, Central South University, Changsha, Hunan, China; ^4^ Department of Experimental Radiation Oncology, The University of Texas MD Anderson Cancer Center, Houston, TX, United States

**Keywords:** IBSP, pan-cancer, prognosis, immunotherapy, osteosarcoma

## Abstract

**Background:**

IBSP is a member of the small integrin-binding ligand N-linked glycoprotein (SIBLING) family that plays a vital role in bone formation, renewal and repair. Emerging evidence revealed that IBSP participated in the tumorigenesis and progression in some cancers. However, its significance in tumour prognosis and immunotherapy is still unknown.

**Methods:**

In the current study, we studied the role of IBSP in tumorigenesis, tumor diagnosis, genomic heterogeneity, methylation modifications, immune infiltration, and therapy response in pan-cancer. In addition, we constructed a risk score model to assessed the prognostic classification efficiency of IBSP using the co-expression genes of IBSP in osteosarcoma (OS), and analyzed the expression and role of IBSP in OS through a series of assays *in vitro*.

**Results:**

IBSP was upregulated in various cancers compared to the paired normal tissues, and it was strongly correlated with the prognosis, pathological stage, diagnostic accuracy, genomic heterogeneity, methylation modification, immune infiltration, immune and checkpoint. Moreover, the predictive model we established in combination with the clinical characteristics of OS patients showed high survival predictive power in these individuals. The assays in vitro showed that IBSP promoted the proliferation, migration and invasion of OS cells, which further confirmed IBSP’s role in cancers.

**Conclusions:**

Our research revealed the multifunctionality of IBSP in the tumorigenesis, progression and therapy in various cancers, which demonstrated that IBSP may serve as a potential prognostic biomarker and a novel immunotherapy target in pan-cancer.

## 1 Introduction

Osteosarcoma (OS) is the most prevalent primary malignant bone tumor, with an annual incidence of 3-4.5 cases per million individuals worldwide (1), and it occurs most frequently in children and adolescents, followed by the elderly over 60 (2). OS is a highly heterogeneous and aggressive malignancy that is prone to distant metastasis at an early stage, with the lungs being the most common metastasis site (3). Since the 1970s, neoadjuvant chemotherapy has been used clinically extensively, the five-year survival rate of OS patients with minimal lesions has increased dramatically from 20% to over 60% (4). However, the overall survival and prognosis of OS patients, particularly those with distant metastases and postoperative recurrence, haven’t improved since then (5). Therefore, to ameliorate the prognosis and overall survival of OS patients, novel therapy targets are urgently required.

The integrin-binding sialic acid protein (IBSP) gene is located in the q28-q31 region of chromosome 4 (6). As a member of the small integrin-binding ligand N-linked glycoprotein (SIBLING) family, the secreted bone sialoprotein (BSP) encoded by it is the main structural protein of the bone matrix and is involved in the early process of regulating bone mineralization (7, 8). IBSP was initially found to play a role in promoting bone formation, bone renewal and repair in some studies (8), but some recent researches have revealed that the overexpression of IBSP is correlated to a poor prognosis in some cancers, such as breast cancer (9), colon cancer (10), esophageal cancer (11) and renal cell cancer (12). According to these studies, IBSP may be a promising biomarker for prognosis prediction in various cancers. However, no research has explored the pan-cancer analysis of IBSP using systematic multi-omics analysis.

In the current study, we performed a comprehensive pan-cancer analysis of IBSP using different bioinformatics approaches, we focused on the correlation of its overexpression with immune infiltration, epigenetic modifications and prognosis in various cancers. We also verified its role in OS using the experiments *in vitro*. In addition, we constructed a risk score model based on IBSP-related genes for OS patients and validated the model using the data from an external dataset.

## 2 Materials and methods

### 2.1 Data acquisition and differential expression analysis

RNA-Seq data from the Cancer Genome Atlas (TCGA) pan-cancer dataset (10,536 samples) and Genotype-Tissue Expression (GTEx) dataset (7,863 samples) were downloaded from the UCSC Xena (xena.ucsc.edu/). The “Primary Tumor” and “Solid Tissue Normal” data were extracted from the TCGA pan-cancer dataset and visualized by the R software (version 4.2.1). IBSP expression in tumor and normal tissues were explored using the Tumor Immune Estimation Resource 2.0 (TIMER2.0, http://timer.cistrome.org/). IBSP expression in pan-cancer were analyzed using the Sanger (http://sangerbox.com/). IBSP expression in different tumor stages were analyzed using the GEPIA 2 database (http://gepia2.cancer-pku.cn/). The data of OS patients were obtained from the GEO database (https://www.ncbi.nlm.nih.gov/geo/) and the GDC database (https://portal.gdc.cancer.gov/). The RNA-seq and clinical phenotype data about 88 patients were downloaded from the GDC database. The GEO database was searched for datasets containing “OS” and “Homo sapiens”, with the inclusion criteria being the number of samples exceeded 50, the results are shown in GSE21257 (GPL10295). In addition, we obtained two datasets containing OS tissues and normal tissues from the GEO database (GSE16088 and GSE42352).

### 2.2 The correlation between IBSP and prognosis of pan-cancer

The association between IBSP and overall survival or disease-free survival (DFS) in pan-cancer were analyzed using the GEPIA2 database, patients were divided into high and low expression groups based on the median of IBSP expression value in the TCGA pan-cancer dataset. Meanwhile, R software was used to plot the connection between IBSP expression and pan-cancer prognosis, the Kaplan-Meier method was used to analyze overall survival, disease-free interval (DFI), disease-specific survival (DSS) and progression-free interval (PFI).

### 2.3 Genetic alterations of IBSP in pan-cancer

The IBSP mutation types and mutation frequencies of multiple cancers in the cBioPortal database (https://www.cbioportal.org/) were analyzed according to the TCGA pan-cancer dataset (32 cancers, a total of 10,967 samples). The frequency of IBSP mutations in TCGA tumor types was explored in the TIMER2.0 database. The Sanger was used to analyze the relationship between tumor mutation burden (TMB) and microsatellite instability (MSI) and pan-cancer IBSP expression.

### 2.4 Correlation and functional enrichment analyses

The Correlations of IBSP expression with StromalScore, ImmuneScore, and ESTIMATEScores were performed for each TCGA tumor type using the Sanger online site, the results were imported into R software, and radar plots were created using the “ggradar” package. Similarly, the correlation between IBSP and immune cell infiltration levels (Timer), RNA modification genes (m^6^A, m^5^C, m^1^A) and immune checkpoint genes (ICP) in TCGA tumors was analyzed in Sanger.

Eighty-four samples in the TARGET-OS dataset and 53 samples in the GSE21257 dataset had corresponding clinical information, the sample information for the two datasets is shown in **Table S1**. The TARGET-OS dataset and the GSE21257 dataset were calculated separately, and genes with |rho|>0.5 and p<0.05 were extracted as IBSP-related genes using the “spearman” correlation method. The IBSP-related genes were imported into Cytoscape software (version 3.9.1) to plot the IBSP-related gene network. In the Metascape database (https://metascape.org), investigations of Gene Ontology (GO) and Kyoto Encyclopedia of Genes and Genomes (KEGG) pathway enrichment were carried out. The Venn diagrams were plotted to extract the intersection of IBSP-related genes in the TARGET-OS dataset and the GSE21257 dataset.

### 2.5 Establishment and evaluation of predictive models in OS

The TARGET-OS dataset was used as the training set and the GSE21257 dataset was used as the external validation set. The univariate Cox analysis was performed on the IBSP-related genes shared by the two datasets, and the genes exhibiting significant differences in the univariate Cox analysis were screened using the Least Absolute Shrinkage and Selection Operator (LASSO) regression, and the risk score model was established based on the sum of the lasso regression coefficients and the product of gene expression. The median risk score value was used as the threshold of the high-risk and low-risk groups. Nomogram were constructed by combining the clinical characteristics of OS patients, and the model’s predictive power was evaluated using calibration curves and time-dependent ROC curves.

The “estimate” package (13), “GSVA” package (14) and “IBOR” package (15) were used to calculate the ESTIMATE score, estimate the degree of immune cell infiltration for the high and low-risk groups. The correlation of risk score with ICP genes and ligand genes was analyzed in the TARGET-OS dataset by the “spearman” method, all ICP and ligand genes are shown in **Table S2**. Assessing the potential clinical efficacy of immunotherapy in different risk groups through the Tumor Immune Dysfunction and Exclusion online websites (TIDE, http://tide.dfci.harvard.edu/).

### 2.6 Cell culture and cell transfection

Human normal osteoblast (hFOB), normal liver cells (L-02), esophageal epithelial cells (H031), lung epithelial cells (BEAS-2B), renal epithelial cell (H193), breast epithelial cells (MCF 10A), lung epithelial cells (HCoEpiC) and all the cancer cell lines were obtained from Xiangya Medical College Cell Bank (Changsha, China). Saos-2 cells were cultured in McCoy’s 5A medium (Gibco, Waltham, MA, USA), and the others were cultured in Dulbecco’s Modified Eagle Medium (Gibco) with 10% FBS (Gibco). shRNAs were obtained from Genechem (Shanghai, China). The cells were transfected according to the recommended protocol, and screened using 2 ug/mL puromycin (Beyotime, China). The sequences of shRNA-IBSP were as follows:

shIBSP-1, forward 5’-GCCUAUGAAGAUGAGUACA-3’,
reverse 5’-UGUACUCAUCUUCAUAGGC-3’;
shIBSP-2, forward 5’-GGCACCUCGAAGACAACAA-3’,
reverse 5’-UUGUUGUCUUCGAGGUGCC-3’;
negative control (shNC), forward 5’-UUCUCCGAACGUGUCACGU-3’,
reverse 5’- ACGUGACACGUUCGGAGAA-3’.

### 2.7 Quantitative real-time PCR

PrimeScript RT kits (TaKaRa, Japan) were used for biosynthesis of cDNA. SYBR Premix ExTaq (TaKaRa, Japan) were used for qPCR. The detailed steps are carried out according to the recommended protocol. mRNA primers were as follows:

IBSP, forward 5’-AACAAGGCATAAACGGCACCAGTA-3’,
reverse 5′-CGGTAATTGTCCCCACGAGGTT-3′;
GAPDH, forward 5′-CGGGAAGCTTGTCATCAATGG-3,
reverse 5′-GGCAGTGATGGCATGGACTG-3′.

### 2.8 Western blot analysis

The protein extraction and western blotting procedures used have been described in our previous study (16). The antibodies were as followed: GAPDH (1:2000; Cell Signalling Technologies, Danvers, MA, USA), IBSP (1:1000; Proteintech, Rosemont, IL, USA), MMP2 (1:1000; Proteintech) and MMP9 (1:1000; Proteintech).

### 2.9 Immunohistochemistry, immunofluorescence, CCK-8, wound healing assay, transwell assay and colony formation assay

The experimental procedure used has been described in detail in a previous study (16). The antibodies were as followed: IBSP (1:100; Proteintech).

### 2.10 Statistical analysis

All experiments *in vitro* were repeated three times independently. The results were reported as mean standard deviation, and the differences between the non-normally distributed variables were assessed using the Wilcoxon rank-sum test, which was performed using GraphPad Prism 9.0 and R software. Significance of differences between groups was assessed using Student’s t-test (p < 0.05 indicates statistical significance).

## 3 Result

### 3.1 IBSP is overexpression in pan-cancer

**Figure 1:**
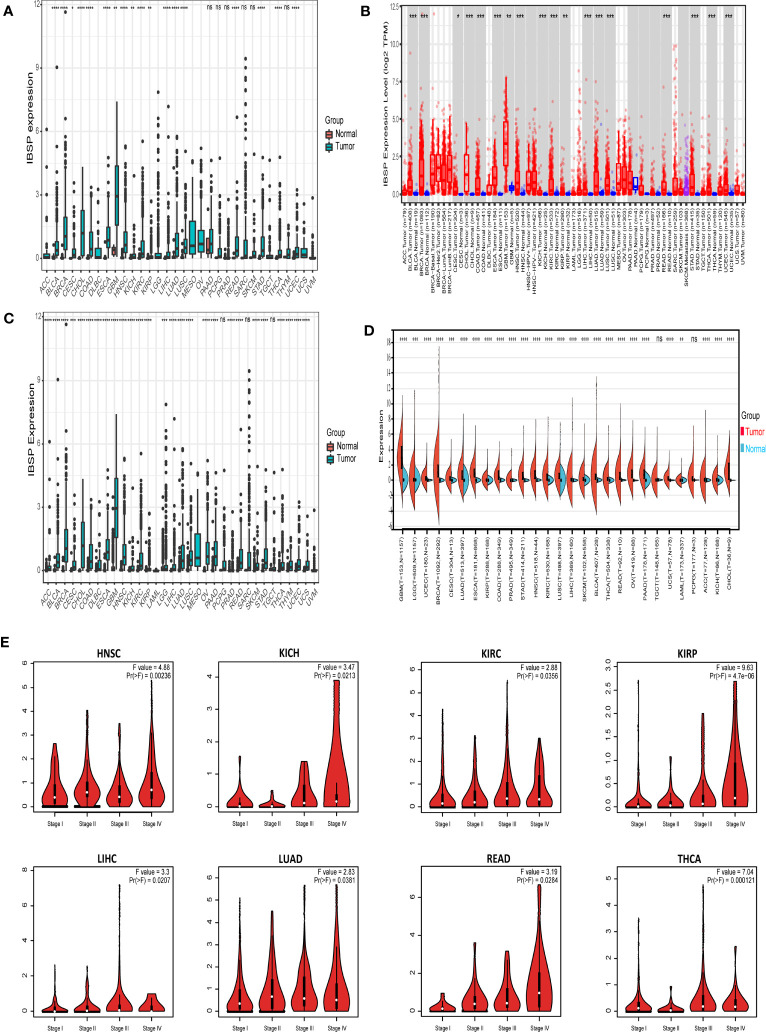
Different expression of IBSP in pan-cancer. **(A)** The mRNA expression of IBSP from the TCGA datasets. **(B)** The mRNA expression of IBSP from the TIMER database. **(C)** The mRNA expression of IBSP from the TCGA and GTEx datasets. **(D)** The mRNA expression of IBSP from the TCGA and GTEx datasets were analyzed using the Sanger. **(E)** The mRNA expression of IBSP in different tumor stages were analyzed in the GEPIA2 database. *P < 0.05, **P < 0.01, ***P < 0.001, ****P < 0.0001. ns, no significance.

The RNA-Seq analysis from TCGA revealed IBSP was overexpressed in 18 types tumors including breast invasive carcinoma (BRCA), bladder urothelial carcinoma (BLCA), cervical squamous cell carcinoma (CESC), colon adenocarcinoma (COAD), glioblastoma multiforme (GBM), cholangiocarcinoma (CHOL), esophageal carcinoma (ESCA), head and neck squamous cell carcinoma (HNSC), kidney renal papillary cell carcinoma (KIRP), kidney renal clear cell carcinoma (KIRC), kidney chromophobe (KICH), uterine corpus endometrial carcinoma (UCEC), liver hepatocellular carcinoma (LIHC), lung adenocarcinoma (LUAD), lung squamous carcinoma (LUSC), stomach adenocarcinoma (STAD), thyroid cancer (THCA) and rectal adenocarcinoma (READ) (**Figure 1A**). Analysis of IBSP expression in the TIMER2.0 database further validated our finding (**Figure 1B**). Combined analysis of TCGA and GTEx datasets, IBSP is also overexpressed in diffuse large B-cell lymphoma (DLBC), adrenocortical carcinoma (ACC), ovarian serous cystadenocarcinoma (OV), pancreatic adenocarcinoma (PAAD), brain Lower Grade Glioma (LGG), uterine carcinosarcoma (UCS), skin cutaneous melanoma (SKCM), thymoma (THYM), and prostate adenocarcinoma (PRAD) (**Figures 1C, D**). We further investigated the association between IBSP and the pathological stage in each tumor, the result revealed that elevated IBSP expression indicated advanced pathological stages in the following tumor types: KIRC, HNSC, KIRP, LUAD, KICH, LIHC, THCA and READ (**Figure 1E**). Moreover, the ROC curve analysis of the TCGA pan-cancer dataset revealed that IBSP had high diagnostic accuracy in CESC, BRCA, COAD, CHOL, HNSC, ESCA, LUSC, STAD and GBM; Moderate diagnostic accuracy was found in KICH, BLCA, LIHC, KIRC, PCPG, UCEC, LUAD, THCA, SARC and READ; Low diagnostic accuracy in KIRP, PAAD, PRAD, and THYM (**Figure S1**). These results suggested that IBSP was upregulated in various cancers, and it may be used as a biomarker for the diagnosis of cancers.

### 3.2 IBSP correlates with the prognosis of multiple tumors

**Figure 2:**
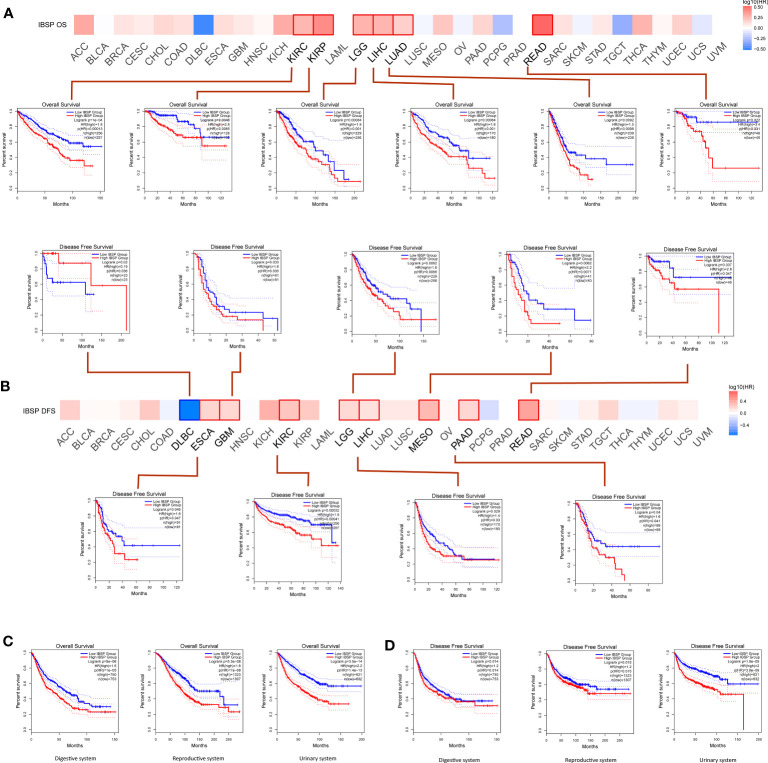
Effects of IBSP expression on the prognosis of pan-cancer. **(A)** Effects of pan-cancer IBSP expression on overall survival. **(B)** Effects of pan-cancer IBSP expression on disease-free survival. **(C)** Effects of IBSP expression on overall survival in digestive system tumors, reproductive system tumors and urinary system tumors. **(D)** Effects of IBSP expression on disease-free survival in digestive system tumors, reproductive system tumors and urinary system tumors.

Analysis of the GEPIA2 database suggested the overexpression of IBSP significantly reduced the overall survival in KIRC, KIRP, LIHC, LGG, READ and LUAD (**Figure 2A**). Additionally, IBSP overexpression decreased patients’ DFS in ESCA, GBM, KIRC, LGG, LIHC, mesothelioma, PAAD and READ (**Figure 2B**). According to the classification of physiological system, COAD, CHOL, LIHC, ESCA, STAD, READ, PAAD, and PAAD belongs to the digestive system cancers, UCS, UCEC, TGCT, PRAD, OV, and CESC belongs to the reproductive system cancers, BLCA, KICH, KIRC, and KIRP belongs to the urinary system cancers, then survival analysis was performed in the GEPIA2 database according to this new classification. The results indicated that patients with IBSP overexpression in digestive system tumors, reproductive system tumors and urinary system tumors had shorter overall survival (**Figure 2C**). Similarly, patients with high IBSP expression had shorter DFS in digestive system tumors, reproductive system tumors and urinary system tumors (**Figure 2D**). Finally, survival analysis of the TCGA pan-cancer datasets revealed that IBSP overexpression was linked to a shorter overall survival, DFI, DSS and PFI in multiple cancers (**Figures S2A–D**). These data suggested IBSP overexpression is correlated to a poor prognosis in multiple cancers.

### 3.3 The mutational landscape of IBSP in pan-cancer

**Figure 3:**
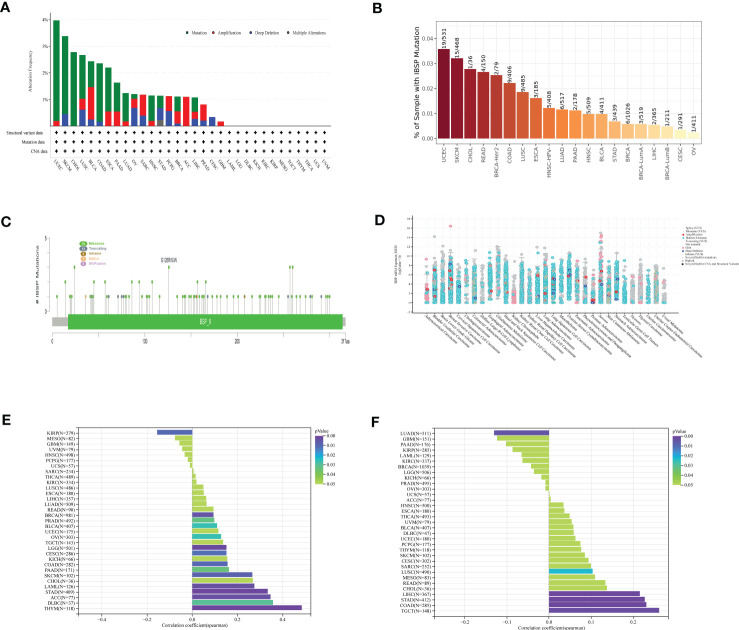
The mutational landscape of IBSP in pan-cancer. **(A)** The genetic alterations of IBSP in the cBioPortal database. **(B)** The genetic alterations of IBSP in The TIMER2.0 database. **(C)** The mutant sites and mutations types of IBSP. **(D)** The counts of IBSP mutation in the pan-cancer from the TCGA dataset. **(E, F)** The relationship between IBSP and TMB/MSI of pan-cancer were analyzed by the Sanger.

The cBioPortal database was analyzed for the genetic alterations of IBSP, the results indicated that IBSP alterations occurred in 20 types cancer in total, among which UCEC having the highest level of alterations, along with “mutation” was the main type, while in BLCA and OV respectively, “amplification” and “deep deletion” were the main types (**Figure 3A**). Then the analysis of the Timer2.0 database showed that IBSP mutations occurred in 16 tumors, among which UCEC also having the highest level of mutation (**Figure 3B**). In addition, A total of 91 mutations sites were detected in IBSP, among which “missense” was the predominant type, followed by “truncating mutations” (**Figure 3C**). We also analyzed the TCGA pan-cancer dataset for the counts of IBSP mutation in the tumors, the result showed widespread genetic alterations of IBSP (**Figure 3D**). Furthermore, we analyzed the association between the IBSP mutation and the clinical outcomes of different tumors (**Figures S3A-R**), only PRAD patients with IBSP mutation had poor prognosis in overall survival (p = 9.320e-4) (**Figures S3R**). The relationship between IBSP and TMB/MSI of the tumors were analyzed by the Sanger, the results revealed that IBSP was positively related to TMB in 13 tumors and negatively related to KIRP (**Figure 3E**), and it was positively related to MSI in LUSC, STAD, COAD, LIHC and TGCT, and negatively related to LUAD (**Figure 3F**).

### 3.4 IBSP is associated immune infiltration and RNA modifying molecules in multiple tumors

**Figure 4:**
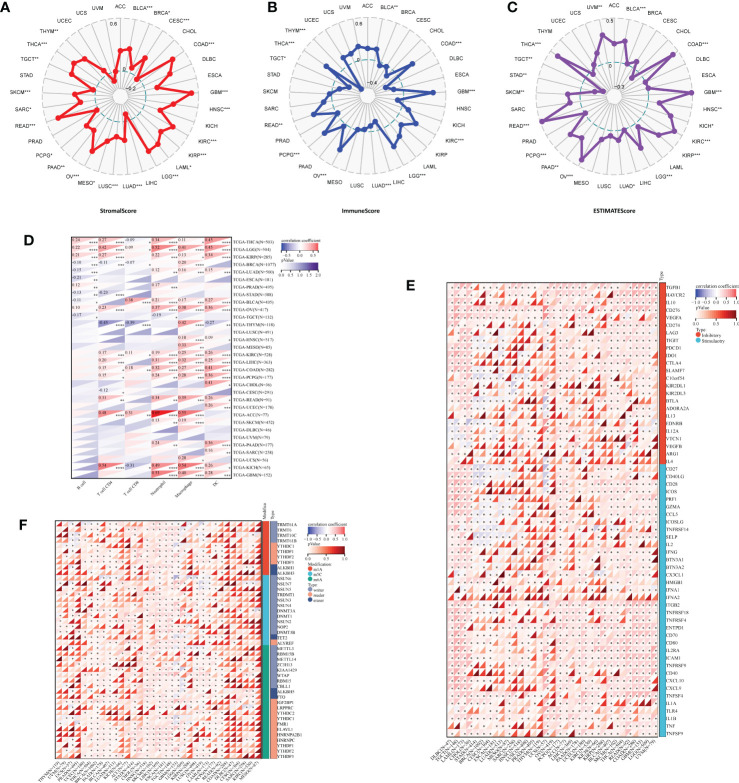
Correlation analysis for IBSP expression with immune cell infiltration levels, immune checkpoints, and RNA modification-related molecules. **(A–C)** The correlation between IBSP expression and StromalScore, ImmuneScore and ESTIMATEScore. **(D)** The correlation between IBSP expression and tumor infiltration levels was analyzed in the TIMER database. **(E)** Pan-cancer co-expression analysis for IBSP and immune checkpoint genes (ICP). **(F)** Co-expression analysis for IBSP and RNA modification-related molecules. *P < 0.05, **P < 0.01, ***P < 0.001, ****P < 0.0001.

Data analyzed by the Sanger suggested that IBSP in STAD and TGCT were negative correlated with ESTIMATEScores, which suggested that IBSP overexpression was associated with reduced stromal cell and immune cell in both tumors, resulting in higher tumor purity. However, in BLCA, COAD, GBM, HNSC, KICH, KIRC, KIRP, LGG, LUAD, OV, PAAD, PCPG, READ, SKCM, THCA and UVM, IBSP expression was positively correlated with the ESTIMATEScores (**Figures 4A–C**). Previous studies have verified that immune cell infiltration and RNA modification molecules are closely related to tumor development, metastasis and prognosis (17, 18). Therefore, we assessed the correlation of IBSP with pan-cancer immune cell infiltration and RNA modification molecules using the Sanger, the result suggested that IBSP was related to all six types of immune cells in LGG, TGCT, KIRP, KIRC, and BLCA (**Figure 4D**). We further explored the relationship between IBSP and 60 immune checkpoint genes (including 36 stimulatory and 24 suppressive genes), and found a substantial relationship between IBSP and five immune-suppressive checkpoint genes (TGFB1, HAVCR2, IL10, CD276, and VGEFA) in most tumors (**Figure 4E**). Meanwhile, the analysis on the relationship between IBSP and RNA modification showed that it was also substantially associated to three main types of RNA modification-related molecules (m^6^A, m^5^C and m^1^A) in HNSC, SKCM, LIHC, ESCA, and TGCT (**Figure 4F**).

### 3.5 Enrichment and survival analysis of IBSP in OS

**Figure 5:**
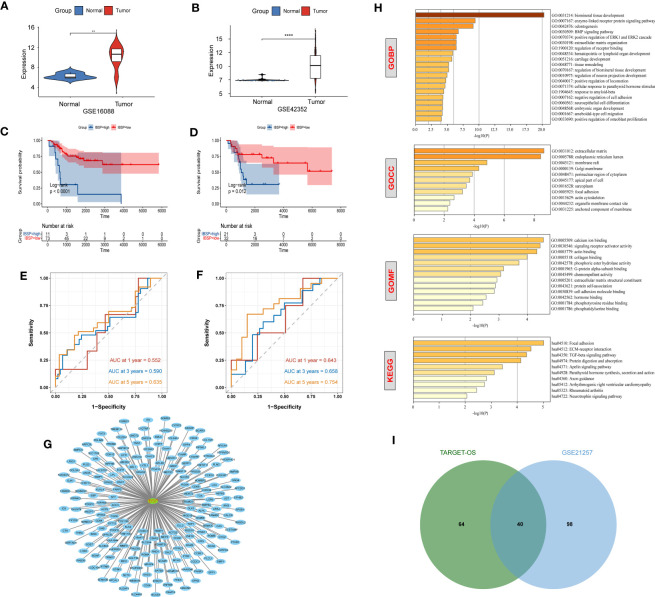
Enrichment and survival analysis of IBSP in OS. **(A, B)** The mRNA expression of IBSP from the GSE16088 and GSE42352 datasets. **(C, D)** Effects of IBSP expression on overall survival in the TARGET-OS and GSE21257 datasets. **(E, F)** The prediction accuracy of IBSP for the 1/3/5 years survival rates of OS patients in the TARGET-OS and GSE21257 datasets. **(G)** A network diagram of IBSP-related genes in the TARGET-OS and GSE21257 datasets. **(H)** GO and KEGG functional enrichment analysis of the molecules interacted with IBSP. **(I)** 40 intersection genes were screened from the TARGET-OS dataset and the GSE21257 dataset. **P < 0.01, ****P < 0.0001.

The aforementioned analysis showed that IBSP may be a potential tumor prognostic marker in various cancers. While, IBSP serve as an indicator of cancer bone metastasis (9, 19–23), its role and mechanism in the primary bone malignancies is still unknown, so we attempted to study its expression and function in OS. In the GSE16088 dataset and GSE42352 dataset, IBSP was significantly over-expressed in OS tissues than that in the paired normal tissues (**Figures 5A, B**). Moreover, OS patients with higher IBSP expression had shorter survival (**Figures 5C, D**). In the TARGET-OS dataset, the prediction accuracy of IBSP for the 1/3/5 years survival rates of OS patients was 0.552, 0.590, and 0.635 respectively (**Figure 5E**). In the GSE21257 dataset, the prediction accuracy of IBSP for the 1/3/5 years survival rates of OS patients was 0.643, 0.658, and 0.754 respectively (**Figure 5F**). It suggested that similar to other tumors, IBSP was a prognostic biomarker in OS, and it can predict patients’ survival. To analyze the biological processes and signaling pathways of the IBSP co-expressed genes in OS, we performed correlation analysis on the two datasets, and 104 IBSP-related genes from the TARGET-OS dataset and 138 IBSP-related genes from the GSE21257 dataset were merged and imported into the Cytoscape software to plot a network diagram of IBSP-related genes (**Figure 5G**). The Enrichment analysis of the IBSP-related genes was performed through the Metascape dataset. GO biological process (GO-BP) indicated that they were related to biomineral tissue formation, enzyme-linked receptor protein signaling pathway, tooth formation, and BMP signaling pathway. GO cell components (GO-CC) indicated that they were related to extracellular matrix and endoplasmic reticulum cavity. GO molecular function (GO-MF) showed that they were related to calcium ion binding, signal receptor activator activity and actin binding. KEGG enrichment analysis revealed that they were correlated to ECM-receptor interaction, local adhesion, TGF-β signaling pathway (**Figure 5H**). Finally, 40 intersection genes were screened from the TARGET-OS dataset and the GSE21257 dataset for subsequent analysis (**Figure 5I**).

### 3.6 Construction and evaluation of OS prediction model

**Figure 6:**
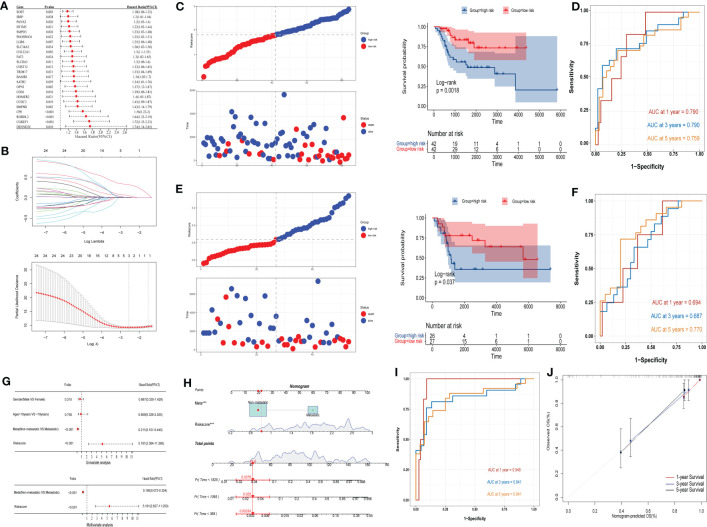
Construction and evaluation of OS prediction model. **(A, B)** Cox analysis and LASSO regression analysis were used to screen out the IBSP-related genes in the TARGET-OS dataset. **(C)** OS prediction model evaluated the overall survival in the high and low risk groups in the TARGET-OS dataset. **(D)** OS prediction model evaluated the accuracy for the 1/3/5 years survival rates of OS patients in the TARGET-OS. **(E)** OS prediction model evaluated the overall survival in the high and low risk groups in the GSE21257 dataset. **(F)** OS prediction model evaluated the accuracy for the 1/3/5 years survival rates of OS patients in the GSE21257 datasets. **(G)** Univariate and multivariate Cox analyses analyzed the clinical characteristics and risk scores. **(H–J)** The predictive model based on clinical characteristics and risk scores had a high predictive accuracy.

Univariate Cox analysis of the 40 IBSP-related genes was performed in the training set TARGET-OS, the results showed that 24 genes exhibited differences in survival (**Figure 6A**). Then the 24 genes were subjected to the LASSO regression analysis, and three genes (CPE, CGREF1 and SOST) were screened out (**Figure 6B**). The risk score calculation formula was as follows: risk score=0.044*CPE+0.005*SOST+0.308*CGREF1. The relationship between IBSP and the three gene expressions and their risk scores in the TARGET-OS and GSE21257 datasets was evaluated using the “spearman” correlation method, the results revealed that IBSP was positively related to their expression and risk scores (**Figures S4A, B**). The risk scores of different clinical characteristics were calculated in the TARGET-OS dataset, the results showed that Female gender, age under 18, and tumor metastasis are risk factors for reduced survival (**Figures S5A–D**). In the TARGET-OS dataset, higher risk groups had shorter overall survival (**Figure 6C**), and the 1/3/5 years survival prediction accuracy of the risk score were 0.790, 0.790, and 0.759 respectively (**Figure 6D**). While in the GSE21257 dataset, overall survival was also significantly reduced in the higher risk group (**Figure 6E**), and the 1/3/5 years survival prediction accuracy were 0.694, 0.687, and 0.770 respectively (**Figure 6F**). Risk score and tumor metastasis were both identified as independent prognostic factors by univariate and multivariate Cox analyses (**Figure 6G**). A nomogram was plotted based on these two indicators, and the value of AUC was used as a predictive indicator (**Figure 6H**). The results show that the 1/3/5 years survival prediction accuracy of this model are 0.948, 0.841, and 0.841 respectively (**Figure 6I**). The results above showed that the predictive model based on clinical characteristics and risk scores had a high predictive accuracy, and the 1/3/5-year survival predictive calibration curves further validated this model (**Figure 6J**).

### 3.7 Levels of immune infiltration and RNA modification analysis in OS

**Figure 7:**
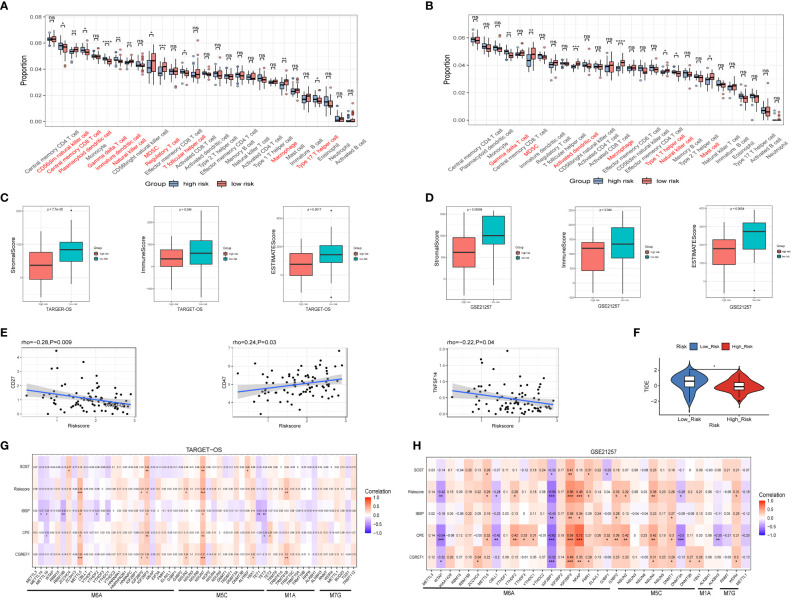
Relationship between IBSP expression and immune infiltration as well as RNA modification. **(A, B)** The levels of immune cells infiltration between the high and low risk groups in the TARGET-OS and GSE21257 datasets. **(C, D)** The StromalScore, ImmuneScore and ESTIMATEScore between the high and low risk groups in the TARGET-OS and GSE21257 datasets. **(E)** Relationship between risk score and ICP gene receptor as well as ICP ligand in the TARGET-OS dataset. **(F)** Analysis of immunotherapy response in the high- and low-risk groups from the TARGET-OS dataset. **(G, H)** The correlation of RNA modifications with IBSP and IBSP-related genes in the TARGET-OS and GSE21257 datasets. *P < 0.05, **P < 0.01, ***P < 0.001, ****P < 0.0001. ns, no significance.

We evaluated the degree of immune cell infiltration in the TARGET-OS and GSE21257 datasets based on the ssGSEA algorithm, the result revealed that the levels of immune cells infiltration between the high and low risk groups is different (**Figures 7A, B**). Furthermore, we analyze immune cell infiltration using the EPIC, CIBERSORT, IPS, MCPCOUNTER, QUANTISEQ, TIMER and XCELL algorithm built into the IBOR package, the similar results are present (**Figures S6A–B**). In both datasets, the ESTIMATEScore of the high-risk group was lower than that of the low-risk group (**Figures 7C, D**), which meant that the cancers in the high-risk group were more purity, and the shorter survival time of the patients may be connected to the level of immune infiltration. In TARGET-OS dataset, risk scores were negatively correlated with ICP gene receptor CD27 and ligand TNFSF14, and positively correlated with ICP gene receptor CD47 (**Figure 7E**). Using the TIDE online website analysis, we found the low-risk group had higher TIDE scores, which suggested that the low-risk group had an increased potential for immune escape and may have a worse response to immunotherapy (**Figure 7F**). Moreover, the correlation of RNA modifications with IBSP and IBSP-related genes (SOST, CGREF1 and CPE) were analyzed in the OS prediction model, the results showed that multiple m^6^A, m^1^A and m^5^C modification genes in the TARGET-OS dataset are closely related to IBSP and IBSP-related genes. In GSE21257 dataset, there are also multiple m^6^A, m^1^A, m^5^C and M^7^G modification genes that are closely related to IBSP and IBSP-related genes (**Figures 7G, H**).

### 3.8 IBSP is highly expressed in OS tissues and cell lines

**Figure 8:**
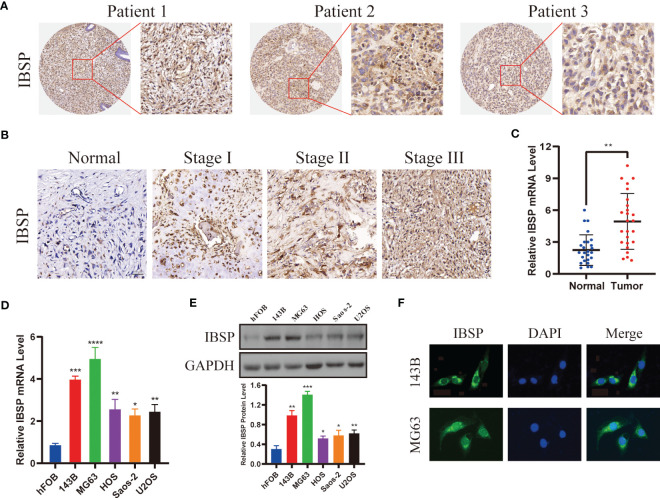
IBSP is overexpressed in OS. **(A)** IBSP expression in the OS tissues was detected by Immunohistochemistry (IHC). **(B)** IBSP expression in the OS tissues at different Enneking stages was detected by IHC. **(C)** IBSP mRNA was detected by qRT-PCR in the OS tissues and peritumor tissues (Normal). **(D, E)** IBSP mRNA and protein were detected by qRT-PCR in the OS cells. **(F)** Cellular localization of IBSP were detected by immunofluorescence (IF). *P < 0.05, **P < 0.01, ***P < 0.001, ****P < 0.0001.

Western blotting, immunohistochemistry, and qPCR assays were used to detected IBSP expression in tumor tissues and different cancer cell lines. Firstly, we detected its expression in several cancers with a high incidence, such as hepatic carcinoma, esophageal cancer, lung cancer, kidney cancer, breast cancer and colorectal cancer. The results showed IBSP was upregulated in these tumor tissues (**Figures S7A–F**). Then we validated its expression in the cancer cell lines, the results of qPCR demonstrated that its mRNA in the different cancer cell lines was elevated compared with the normal cells (**Figures S7G–L**); and the results of WB revealed its protein was overexpressed in the cancer cell lines as well (**Figures S7M–R**). Finally, we proceeded to detected its expression in OS tissues and cell lines. The results showed that OS tissues had higher levels of IBSP expression than the paired normal tissues, and that IBSP expression were elevated in advanced pathological stages (**Figures 8A–C**). qPCR and western blotting were used to detected the expression of IBSP in hFOB and OS cells, and it was upregulated in OS cells, especially in 143B and MG63, thus, they were selected for the further experiments (**Figures 8D, E**). In addition, we also examined the subcellular localization of IBSP in the OS cells, it showed that IBSP was mainly localized in the cytoplasm (**Figure 8F**).

### 3.9 IBSP knockdown suppresses OS proliferation, migration and invasion

**Figure 9:**
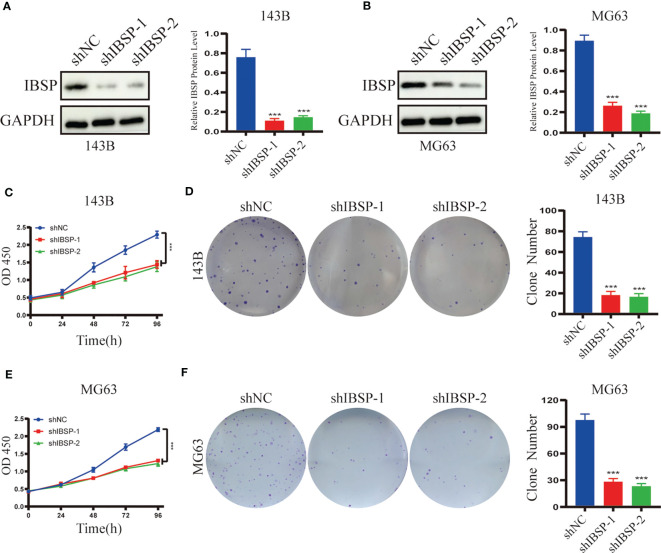
Silenced IBSP inhibits the proliferation of OS cells. **(A, B)** IBSP protein expression was detected in 143B and MG63 cells with IBSP knockdown or control. **(C, D)** Cell proliferation rate was detected by CCK-8 and clone formation assays in 143B. **(E, F)** Cell proliferation rate was detected by CCK-8 and clone formation assays in MG63. ***P < 0.001.

**Figure 10:**
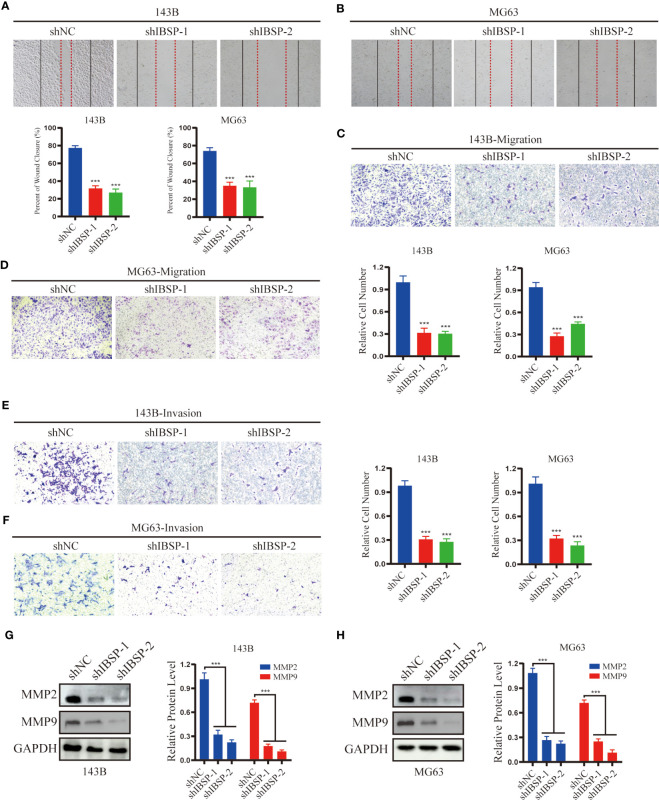
IBSP knockdown suppresses OS cells migration and invasion. **(A, B)** Cell migration rate was evaluated by wound healing assays in 143B and MG63 cells with IBSP knockdown or control. **(C, D)** Cell migration ability was evaluated by transwell assays in 143B and MG63 cells with IBSP knockdown or control. **(E, F)** Cell invasion ability was evaluated by transwell assays in 143B and MG63 cells with IBSP knockdown or control. **(G, H)** The expression of MMP2 and MMP9 was detected in 143B and MG63 cells with IBSP knockdown or control. ***P < 0.001.

To study the role of IBSP on the proliferation of OS, IBSP was knockdown in 143B and MG63 (**Figures 9A, B**). The data of CCK8 and clone formation assays revealed that IBSP knockdown inhibited OS cells proliferation in 143B (**Figures 9C, D**), and MG63 (**Figures 9E, F**). In addition, the transwell assay and the wound healing assay were carried out to clarify the role of IBSP in the metastasis of OS. The wound healing assay indicated that IBSP knockdown would reduce the closure in OS cells (**Figures 10A, B**). The results of transwell assays which included migration and invasion assays, suggested that OS cells permeating the membrane were reduced when IBSP was knocked down (**Figures 10C–F**). MMP2 and MMP9 are commonly used to indicate the metastatic potential of cancer cells. In our study, the expression of MMP2 and MMP9 were significantly reduced when IBSP was knockdown in OS cells (**Figures 10G, H**). The data together revealed that IBSP can promote the migration and invasion of OS cells *in vitro*.

## 4 Discussion

IBSP is a glycoprotein consisting of 301 amino acids, and its terminal Arg-Gly-Asp sequence can bind integrins, which play important roles in cell adhesion, angiogenesis, regulation of extracellular matrix, immune cell migration and infiltrated (24–26). Therefore, some studies have revealed that IBSP promotes bone metastasis of tumor cells in BRCA (9), PRAD (27), and non-small cell lung cancer (21). Recently, with the development of bioinformatics, some studies demonstrated that the expression of IBSP is upregulated in epithelial tumors such as laryngeal cancer (28) and BLCA (29), which is highly correlated with a poor prognosis. For malignant tumors of non-epithelial origin, such as GBM, IBSP can also promote tumor cell proliferation and migration (30). Therefore, IBSP is considered to be an oncogene and closely related to the bone metastasis.

Cancer has become a major threat to human health because of its indistinct symptoms, rapid development, and lack of effective treatment. Therefore, finding biomarkers of tumor progression can be beneficial to early diagnosis and early treatment of cancer patients, which is the main method to improve the efficiency of cancer treatment (31). In this study, based on TCGA data, we found that IBSP was upregulated in various malignant tumor tissues (BRCA, BLCA, CHOL, CESC, ESCA, COAD, KICH, GBM, HNSC, KIRP, KIRC, LUAD, LIHC, LUSC, READ, STAD, UCEC, THCA), and it was found to be significantly associated with poor prognosis of the patients with these malignant tumors. Meanwhile, based on the GTEx dataset, we further confirmed that IBSP was also overexpressed in ACC, DLBC, LGG, OV, PAAD, PRAD, SKCM, THYM and UCS. Furthermore, by analyzing the OS dataset, combined with the results of some assays *in vitro*, we determined that IBSP was overexpressed in OS and associated with a poor prognosis in OS patients.

The extracellular matrix (ECM) is a complex structure made up of numerous proteins and glycans, which was previously considered to act as a barrier for cells, providing mechanical pressure to maintain the normal tissue morphology of cells (32). However, some recent studies have revealed that changes in ECM compositional abundance and structural strength are inseparable to tumor occurrence, development, metastasis and chemotherapy resistance (33). In this study, our data revealed that the interaction genes of IBSP have the function of regulating extracellular matrix, which is consistent with previous studies (10, 27). Moreover, the functional enrichment analysis demonstrated that IBSP and its interaction genes in OS were closely related to biomineral tissue development (GO-BP), extracellular matrix (GO-CC), calcium ion binding (GO-MF), and cell adhesion. Therefore, we speculate that the protein encoded by IBSP binds integrin through its RGD sequence, then recruits calcium ions to reduce the adhesion of OS cells, thereby promoting tumor cell metastasis. In order to verify our conjecture, some cell assays *in vitro* were performed, and the results confirmed that IBSP knockdown would inhibit the migration and invasion of OS cells, and the expression levels of tumor metastasis markers MMP2 and MMP9 were significantly weakened.

The tumor microenvironment contains a variety of immune cells and stromal components, and the occurrence, development, and metastasis of tumors are closely related to changes in the tumor microenvironment (34, 35). Our experiments confirmed that in a variety of tumors, the expression of IBSP was positively correlated with the infiltration of T cells, neutrophils, macrophages and dendritic cells (DC), especially the infiltration of macrophages. Increasing number of evidences showed that tumor-associated macrophages (TAM) are important regulators of tumorigenesis and metastasis, and their high infiltration rate is associated with chemotherapy resistance and poor prognosis (36). Similarly, different levels of immune cell infiltration in OS, including T cells, macrophages, and myeloid-derived suppressor cells, were also present in various risk subgroups, and ESTIMATEscores were lower in the high-risk subgroup than in the low-risk subgroup in both datasets. These differences may all be significant influencing factors resulting in various clinical outcomes in osteosarcoma patients. Immunotherapy is a viable new treatment option to take the place of traditional Chemoradiotherapy because it has demonstrated sufficient efficacy in the management of a range of cancers. Thus, we analyzed the expression of IBSP and ICP genes, and the results showed that IBSP was positively correlated with ICP genes such as VGEFA, HAVCR2, IL10, CD276, and TGFB1 in pan-cancer. In terms of OS, our study demonstrated that patients in the high-risk group not only had shorter survival durations than those in the low-risk group, but also had lower TIDE ratings. Hence, immunotherapy may enable the high-risk group to experience higher therapeutic benefit.

Finally, we contracted a risk score model consisting of three genes (CPE, CGREF1, SOST) that were significantly positively correlated with IBSP expression. Previous studies have confirmed that the three genes CPE, CGREF1, and SOST are closely related to the proliferation, metastasis, and drug resistance of OS (33–35), which imply that IBSP may play a role in the proliferation and migration of OS. Combined with external datasets, the reliability of the risk scoring model was confirmed. After incorporating clinically relevant features, we further established a clinical prediction model, which exhibited high accuracy in survival prediction, which will be helpful for the evaluation and management of OS patients.

However, our study also has some shortcomings. First, we didn’t conduct further studies on the mechanism of IBSP in promoting OS progression; In addition, and we didn’t conduct animal experiments to verify its function *in vivo*; Finally, we didn’t generate IBSP overexpressed OS cells to further verify its role in OS.

In conclusion, we conducted a more comprehensive pan-cancer analysis of IBSP using multi-omics data, and found that IBSP is abnormally expressed in various tumors and highly related to a poor prognosis. We also studied IBSP’s function from the aspects of methylation modification, gene change, immune infiltration, and functional enrichment, which clarified the role of IBSP in tumor development and metastasis. Through bioinformatics methods and experiments *in vitro*, we revealed that IBSP could promote the proliferation and migration of OS cells. Taken together, our study demonstrates that IBSP is a potential prognostic biomarker and immunotherapy target in various tumors including OS.

## Data availability statement

The datasets presented in this study can be found in online repositories. The names of the repository/repositories and accession number(s) can be found within the article/**Supplementary Material**.

## Ethics statement

The study was approved by the ethics committee of the Third Xiangya Hospital, Central South University (Approval No.202023). All animal experiments were in compliance with the Experimental Animal Ethics Committee’s guidelines and were approved by the Animal Experimental Committee of the Third Xiangya hospital (Grant number: 2021sydw0221).

## Author contributions

BP, JC, WT and YD designed the research; BP, JC, XW collected and analyzed the data; JC, RL and YZ performed the research; BP, JH and QF analyzed the data; BP, JC and YZ wrote the paper; BP, XC and JC organized the original source data. All authors contributed to the article and approved the submitted version.

## Supplementary material

Figure S1 The diagnostic sensitivity of IBSP in the TCGA pan-cancer.

Figure S2 The survival analysis of the high and low IBSP expression groups in TCGA dataset, including overall survival **(A)**, disease-free interval **(B)**, disease-specific survival **(C)** and progression-free interval **(D)**.

Figure S3 Effect of IBSP gene mutation on overall survival of various tumors.

Figure S4 The relationship between IBSP and the three gene (CPE, CGREF1 and SOST) in the TARGET-OS **(A)** and GSE21257 **(B)**.

Figure S5 The risk factors for reduced survival in the TARGET-OS dataset **(A)**, which including gender **(B)**, age **(C)** and metastasis **(D)**.

Figure S6 The differences of immune infiltration between the high and low risk groups in the TARGET-OS **(A)** and GSE21257 **(B)** datasets.

Figure S7 The expression of IBSP in various cancer tissues and their cell lines **(A-F)**. The protein level of IBSP was detected in the tumor tissues (T) and adjacent normal tissues (N). **(G-L)** The mRNA of IBSP was detected in different cancer cell lines and their corresponding normal cell lines. **(M-R)** The protein level of IBSP was detected in different cancer cell lines and their corresponding normal cell lines.
