# Supplementary figures and images for: Pan-cancer analysis shows that IBSP is a potential prognostic and immunotherapeutic biomarker for multiple cancer types including osteosarcoma
Source: Front Immunol. 2023 Jun 29;14:1188256. doi: 10.3389/fimmu.2023.1188256 (PMC10339805; doi:10.3389/fimmu.2023.1188256)

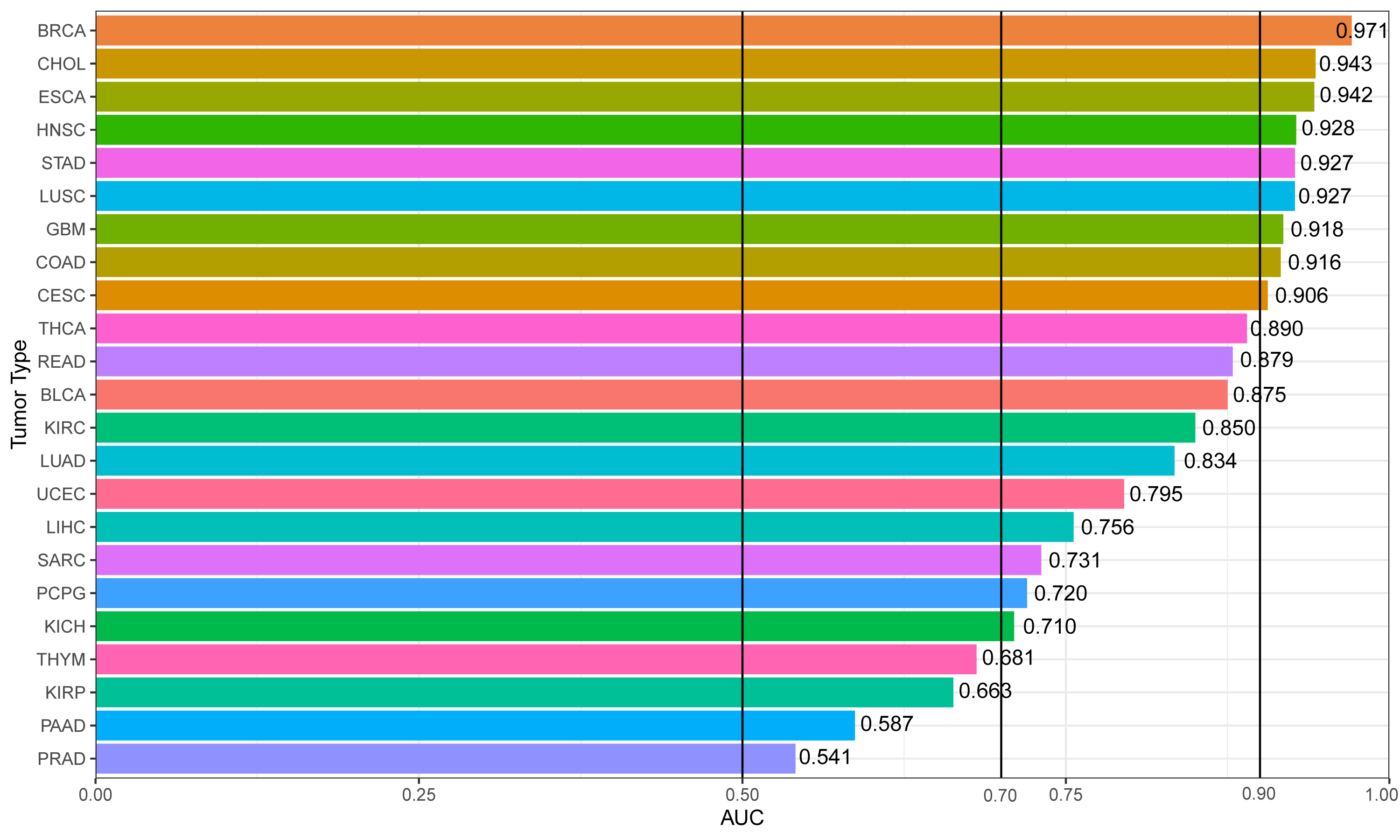

Supplement: Figure S1 — The diagnostic sensitivity of IBSP in the TCGA pan-cancer. [file Image_1.tif]

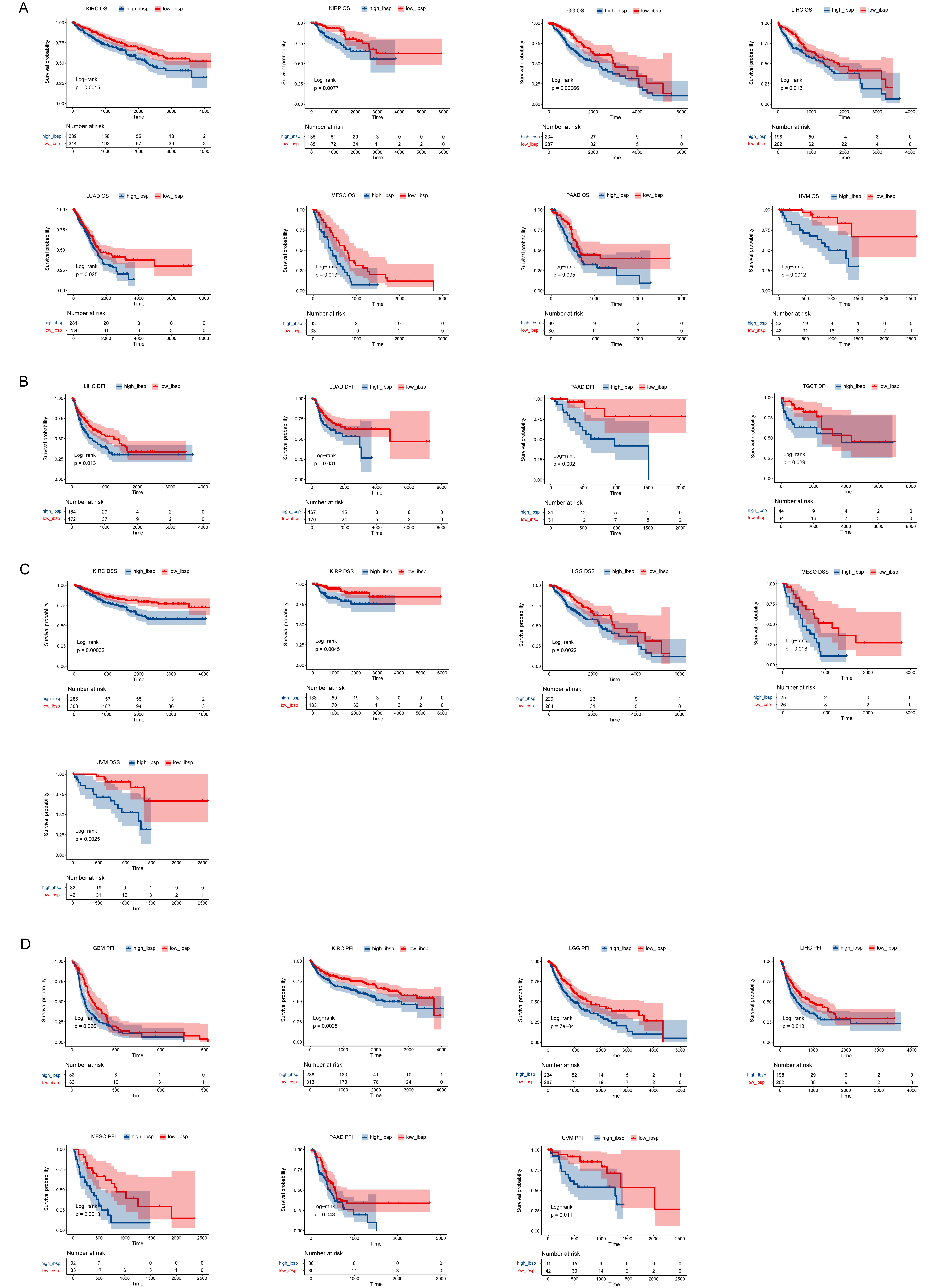

Supplement: Figure S2 — The survival analysis of the high and low IBSP expression groups in TCGA dataset, including overall survival (A), disease-free interval (B), disease-specific survival (C) and progression-free interval (D). [file Image_2.tif]

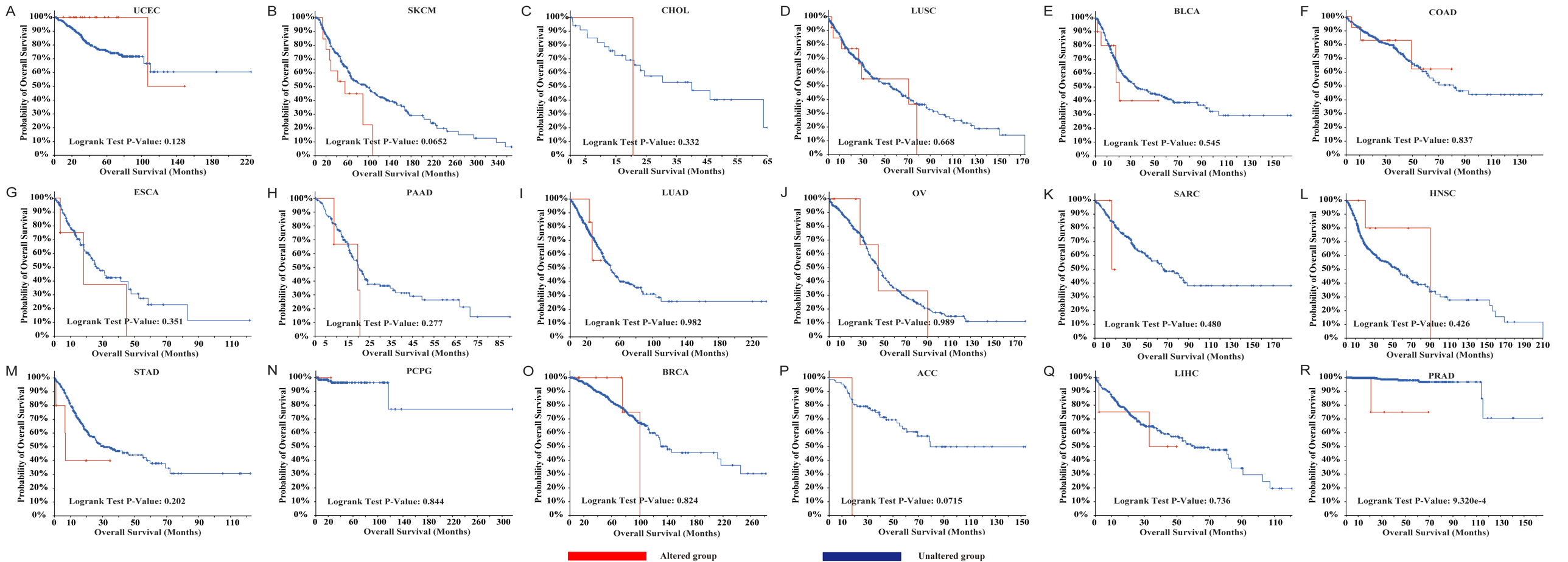

Supplement: Figure S3 — Effect of IBSP gene mutation on overall survival of various tumors. [file Image_3.tif]

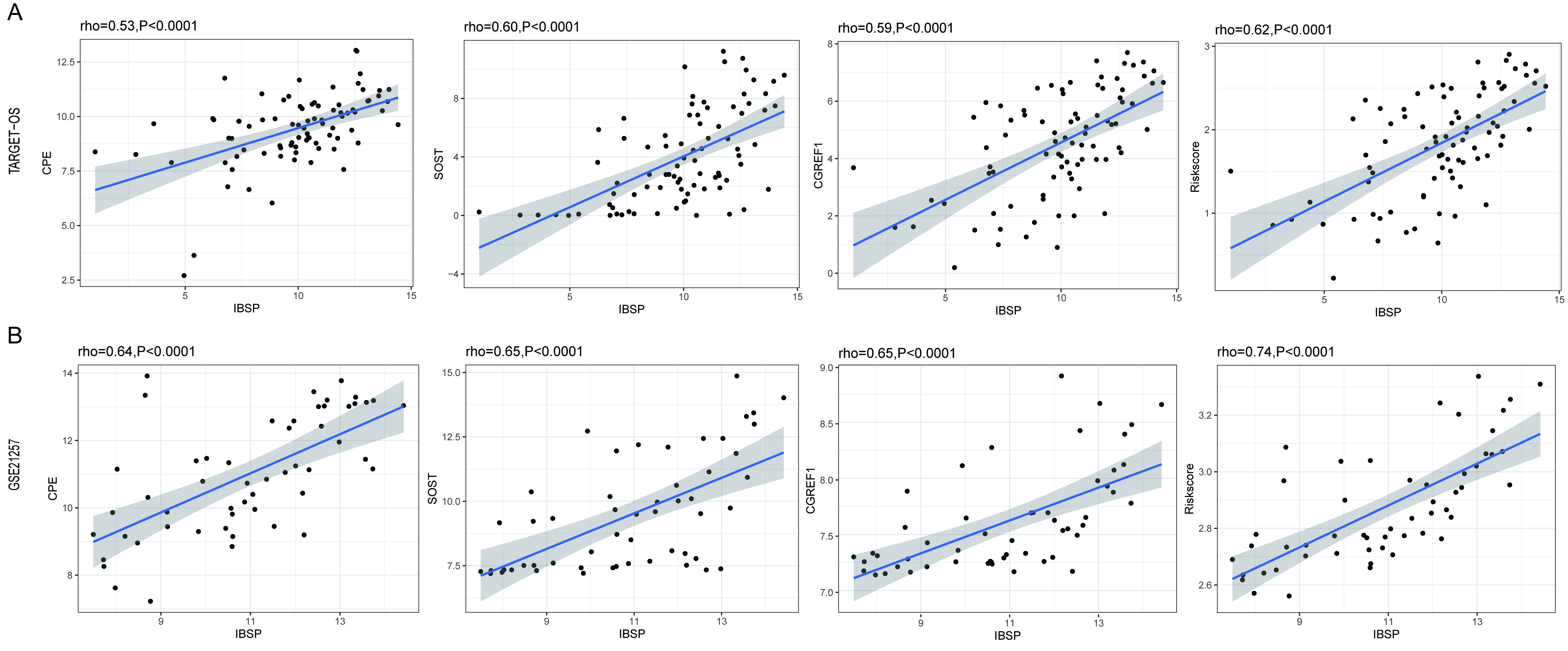

Supplement: Figure S4 — The relationship between IBSP and the three gene (CPE, CGREF1 and SOST) in the TARGET-OS (A) and GSE21257 (B). [file Image_4.tif]

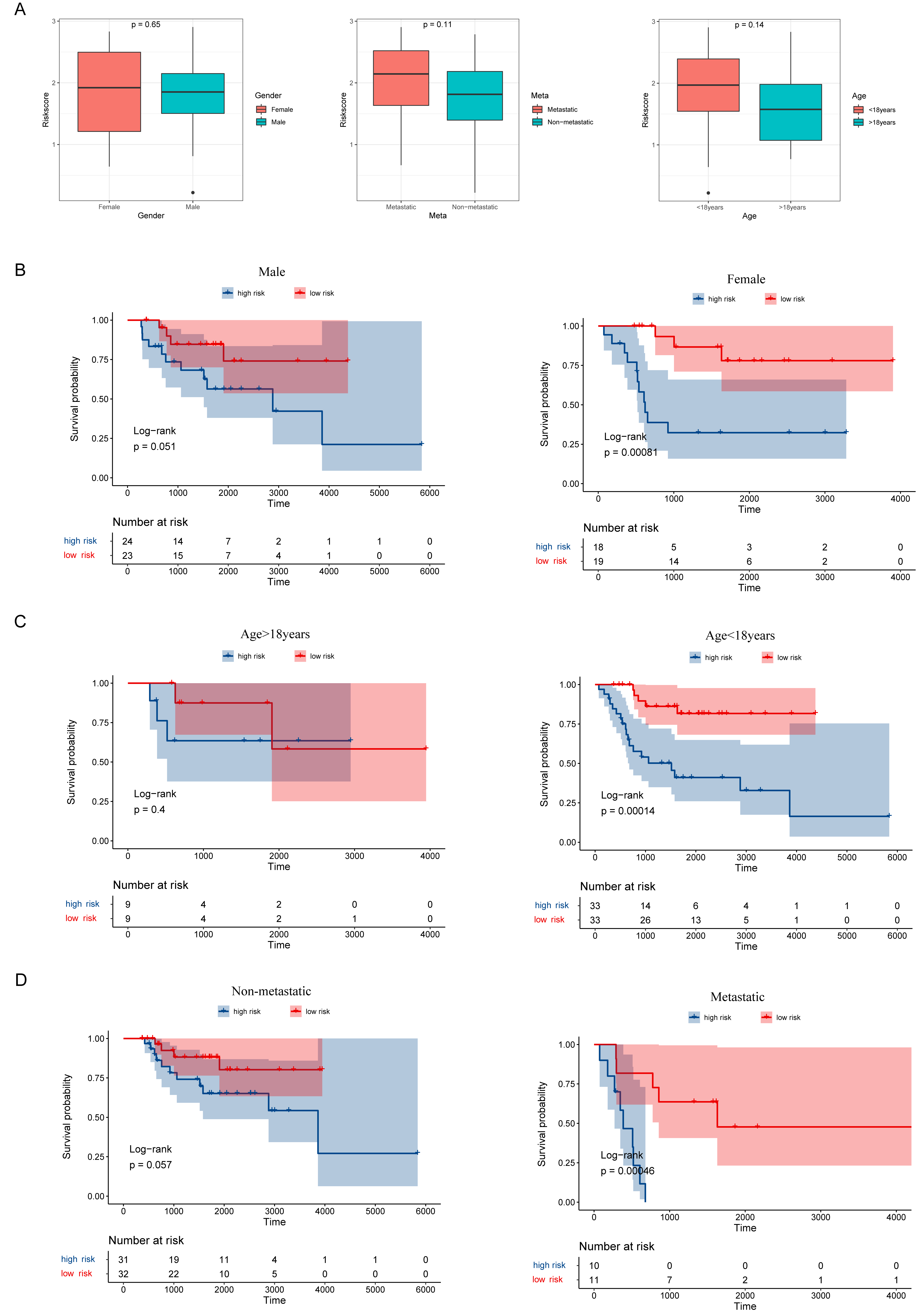

Supplement: Figure S5 — The risk factors for reduced survival in the TARGET-OS dataset (A), which including gender (B), age (C) and metastasis (D). [file Image_5.tif]

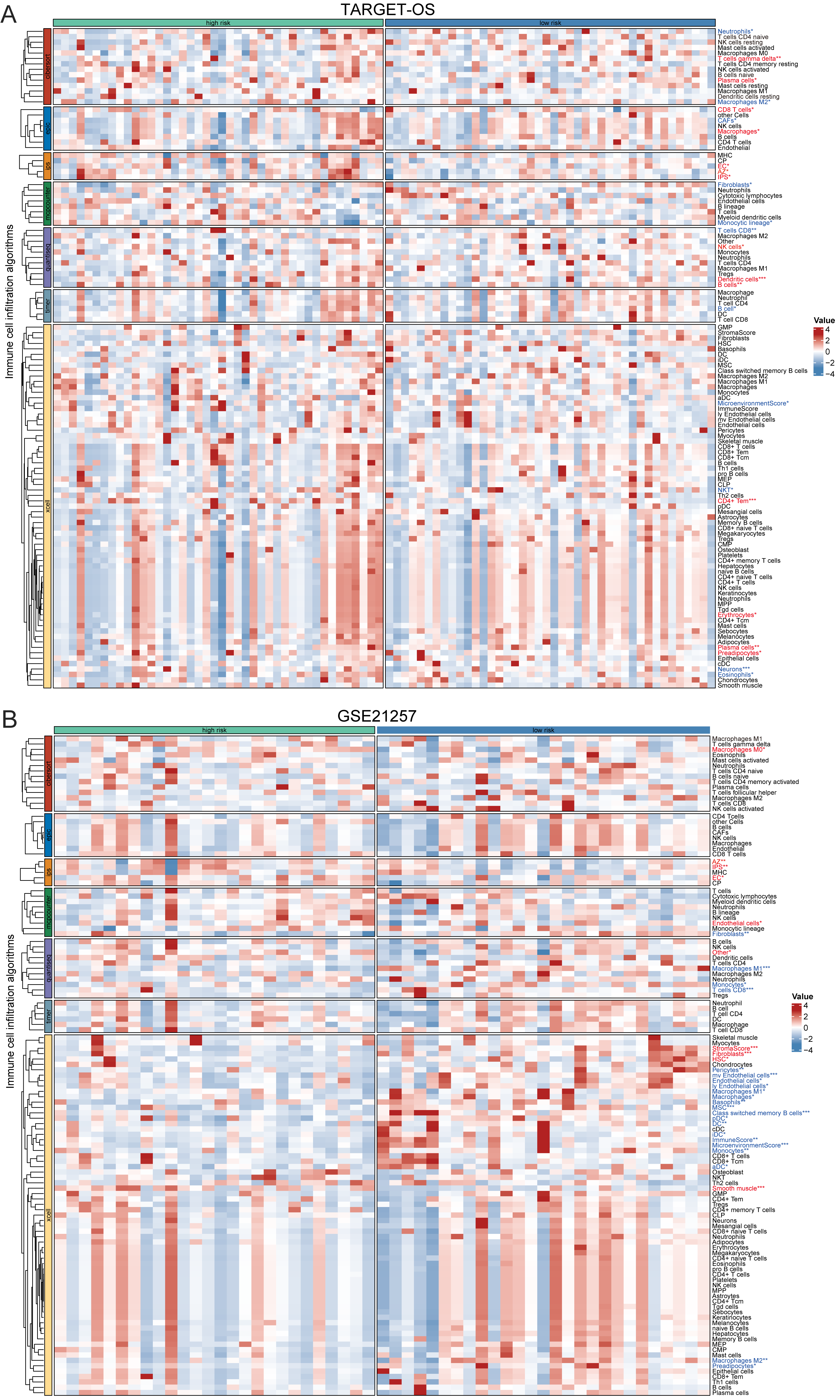

Supplement: Figure S6 — The differences of immune infiltration between the high and low risk groups in the TARGET-OS (A) and GSE21257 (B) datasets. [file Image_6.tif]

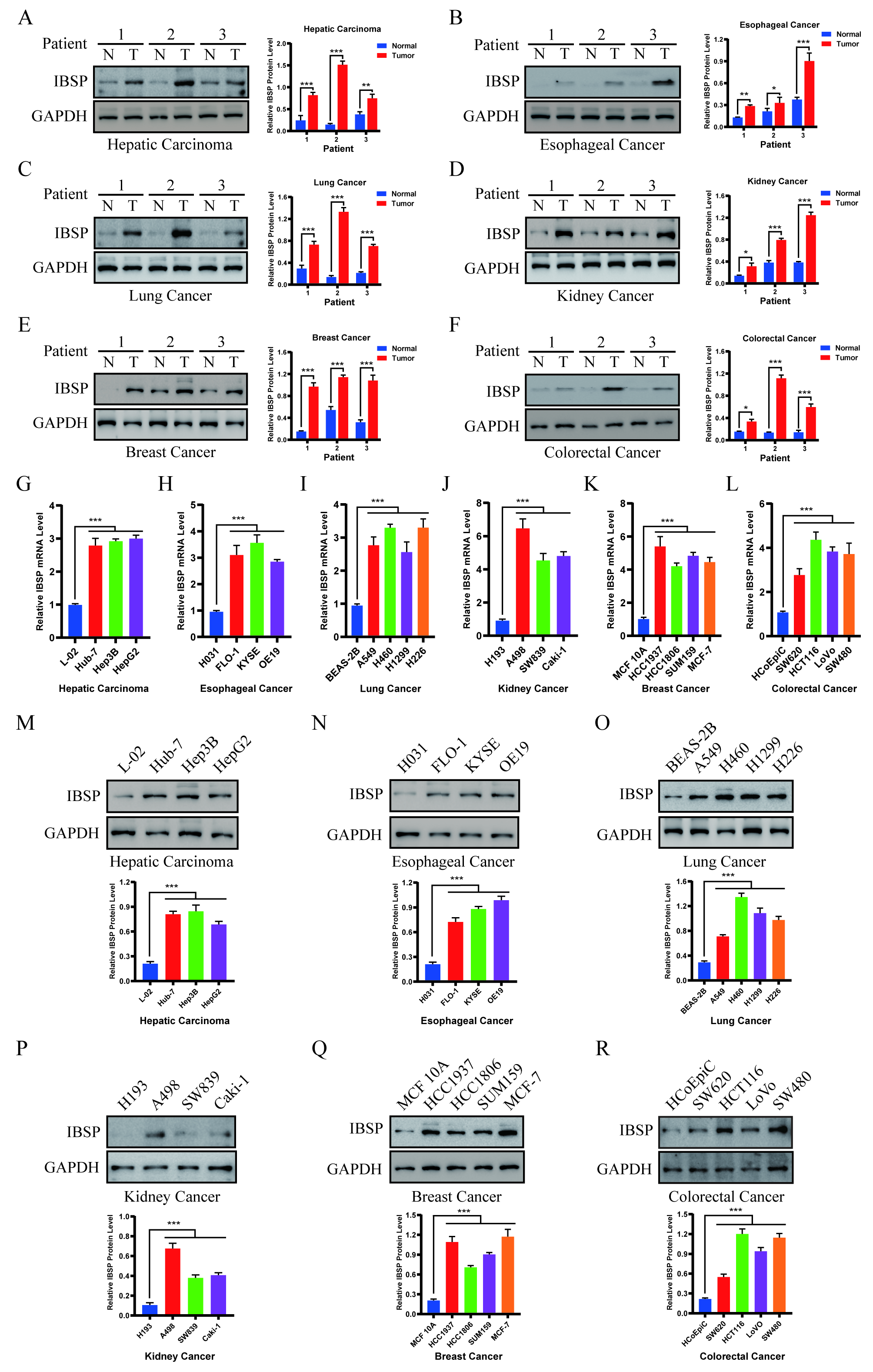

Supplement: Figure S7 — The expression of IBSP in various cancer tissues and their cell lines (A-F). The protein level of IBSP was detected in the tumor tissues (T) and adjacent normal tissues (N). (G-L) The mRNA of IBSP was detected in different cancer cell lines and their corresponding normal cell lines. (M-R) The protein level of IBSP was detected in different cancer cell lines and their corresponding normal cell lines. [file Image_7.tif]
